# An updated reconstruction of basaltic crust emplacement in Tyrrhenian sea, Italy

**DOI:** 10.1038/s41598-017-17625-2

**Published:** 2017-12-21

**Authors:** Carlo Savelli, Marco Ligi

**Affiliations:** 1 Marine Geologist (retired from Consiglio Nazionale delle Ricerche - CNR), Largo G.I. Molina 6, 40138 Bologna, Italy; 2 Ismar-CNR, Via P. Gobetti 101, 40129 Bologna, Italy

## Abstract

Basaltic crust is present in the oceans and marginal seas. Oceanic accretion from inception to ending may be usefully recognized in small basin setting like the Tyrrhenian. Alternating episodes of strong and moderate extensional tectonics characterized the small Tyrrhenian opening. Hyperextension (drifting) of late-Miocene and latemost Pliocene age was followed by Pliocene and Late Quaternary moderate extension (rifting). Early hyperextension (~7.5–6.3 Ma) acted in the submerged margin of Hercynian Sardinia. Sardinia offshore, E-directed low-angle faults were accompanied by MORB-like volcanism of non linear shape in the shallow Vavilov plain - inherited segment of alpine-age orogen. Late hyperextension (~1.9–1.7 Ma) acted along the central N-S lineament of Vavilov plain, former metamorphic core complex. At the lineament northern side, E-dipping detachment faulting exposed serpentinized peridotite. At the other side, Vavilov volcano was faulted and its east flank tilted westwards. At the same time, volcanism with affinity to transitional MORB induced opening of Marsili basin. The drift episodes were characterized by absence or scarcity of volcanic activity on the conjugated emerged margins. The rift episodes (respectively ~5–1.9 Ma, and ~1/0.8 Ma-Recent) saw growth of major north-south trending volcanoes in bathyal area as intense volcanic activity developed on the continental margins.

## Introduction

**Figure 1:**
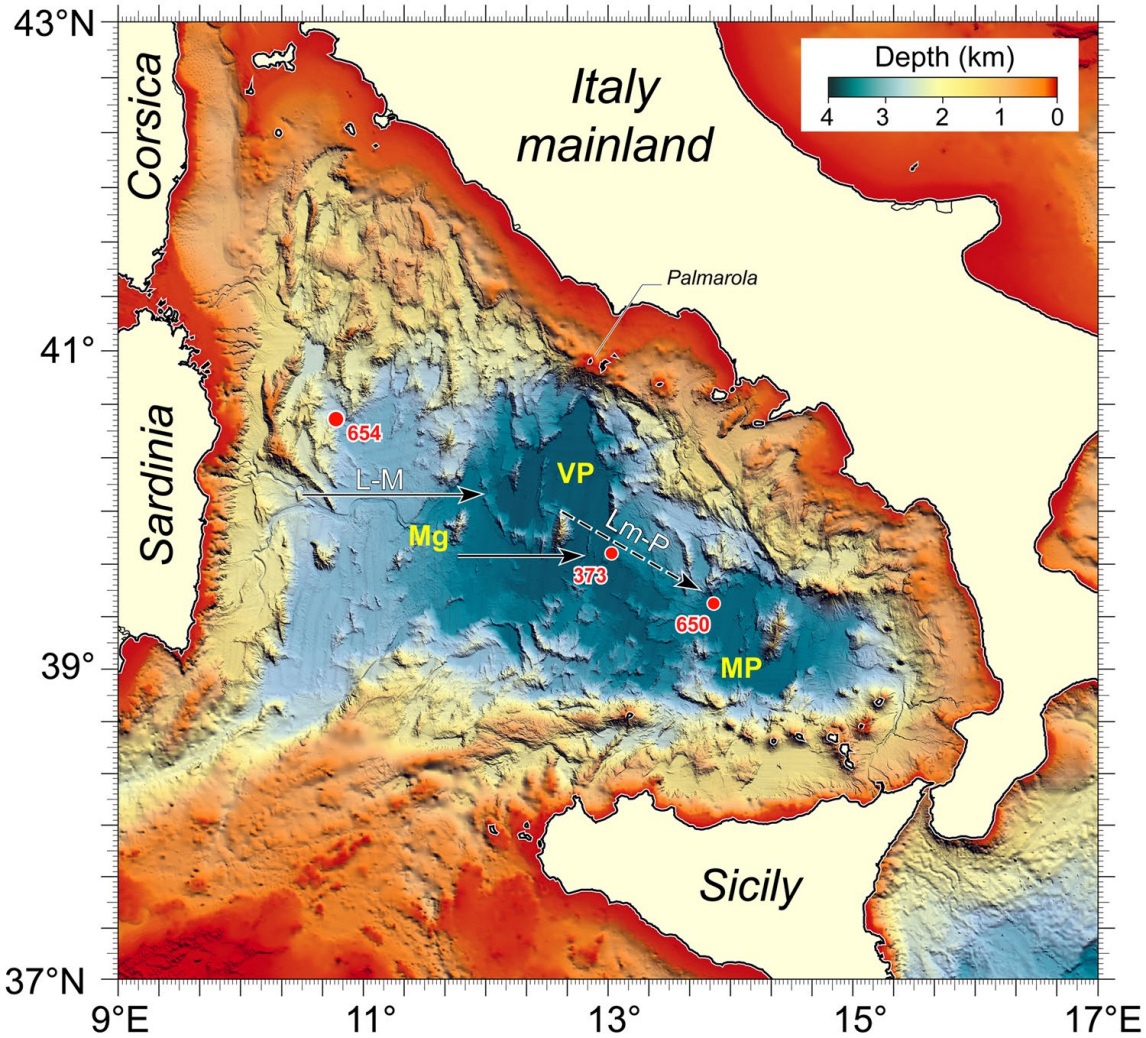
The Tyrrhenian Sea. Shaded relief image derived from bathymetric data with sun illumination from NE, 45° over the horizon and no vertical exaggeration (grid resolution 100 m). Mercator projection at 40° N. Bathymetry of the Tyrrhenian region was obtained by merging all the available multibeam^48–50^ and EMODnet gridded data (http://www.emodnet.eu/bathymetry). The multibeam data were processed by the Kongsberg Neptune/Poseidon packages; spatial analysis and mapping were performed using the GMT^51^ and PLOTMAP^52^ packages. Coastline from different map sources at scale 1:100,000.0 published by Istituto Idrografico della Marina was digitized in geographical coordinates by using the software DIGMAP^53^ and were converted to the WGS84 geodetic datum by the program DATUM^54^. Filled red circles indicate the DSDP 373 and ODP 650 sites that penetrated basaltic crust of Late Miocene (L-M) and Latemost Pliocene (Lm-P) age in the Vavilov and Marsili plains (VP and MP, respectively). Such early oceanization episodes were followed by episodes characterized by the emplacement of corresponding homonymous large central volcanoes. Black arrows indicate the direction of the L-M and Lm-P hyperextension pulses trending E-W and NW-SE, respectively. Besides ODP-Site-650, volcanic manifestations of Lm-P age are found also offshore from Sardinia (ODP-Site-654), and on Palmarola island (see text). Mg = Magnaghi volcano.

**Figure 2:**
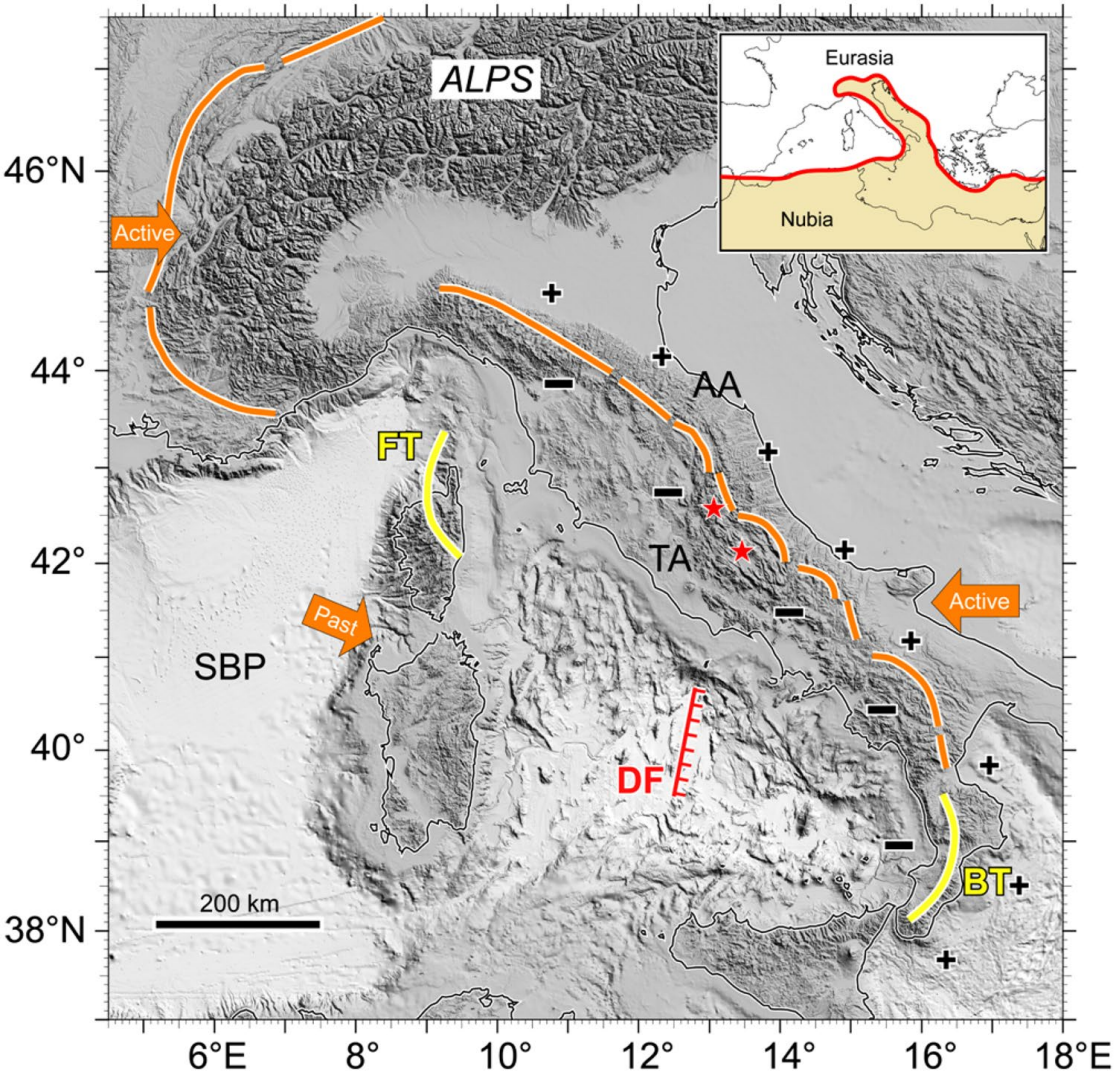
The Alps-Tyrrhenian-Apennines system. Shaded relief image of central Mediterranean region (Mercator projection at 40° N; illumination from NE, 45° over the horizon; horizontal resolution 250 m; no vertical exaggeration). Data^48–50^ and methods^51–54^ as in Fig. 1. Arrows show the east- and west-directed subductions that are present beneath the Alps and Apennines, respectively. Past E-directed descent of European plate to the east of Corsica-Sardinia originated the double vergence of Tyrrhenian submerged orogen of Alpine age. Abbreviations: **AA** = Adriatic-Apennines (external zone of the Apennines); **FT** = Front-thrusts of NE Corsica showing crystalline-metamorphic nature and alpine-age; **TA** = Tyrrhenian-Apennines (internal zone of the Apennines); SBP = Sardinia Basin Provence margin (France); **DF** = East-dipping detachment fault separating front- and back-thrust zones of central Tyrrhenian inherited orogen; **BT** = Back-thrusts of Calabrian Arc showing crystalline-metamorphic nature; **plus** and **minus** signs = extensional (−) and compressional (+) tectonic modes that are present in the Tyrrhenian- and Adriatic-Apennines, respectively; stars = location of Norcia/Amatrice/Monti Sibillini area affected by the extensional earthquake sequence from August 16 to January 2017.

**Figure 3:**
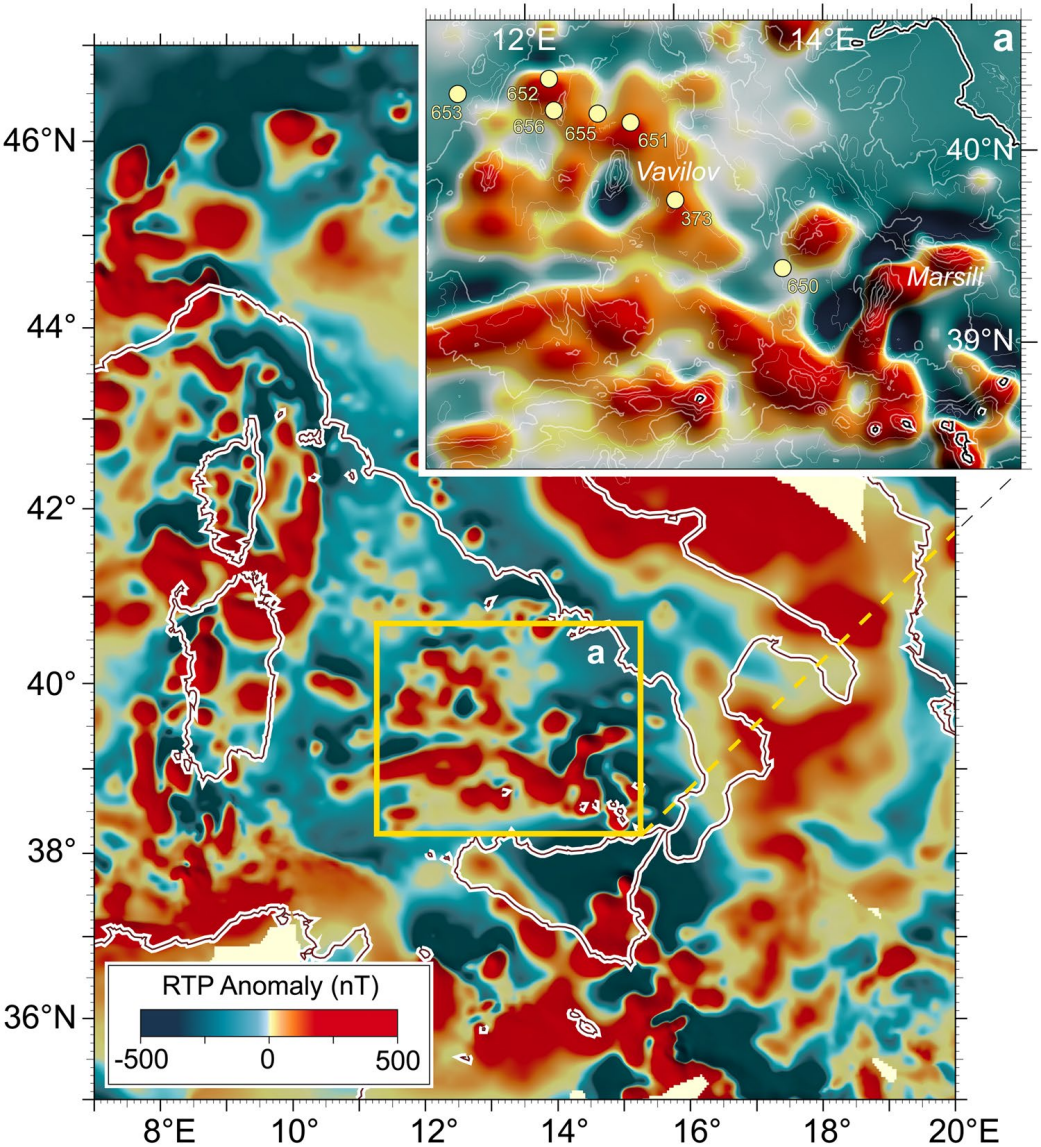
Magnetic anomalies of the Tyrrhenian region. Colour shaded relief map of the reduced to the pole aeromagnetic anomaly field of Italy. The image was obtained with software PLOTMAP^52^ from gridded data of magnetic data projected to the common flight altitude of 2500 m and referred to geomagnetic epoch 1979. Inset (**a**) depicts bathymetry data and N-S trending magnetic lineaments linked to the Vavilov and Marsili volcanoes and surroundings. The anomalies associated with deep-seated basaltic crust have low intensity (up to 200 nT^11^) and shapes which are almost rounded or poorly elongated (diffusional spreading linked to strong extension and low-angle faults; i.e., drill-sites DSDP 373 and ODP 650). By contrast, the magnetic patterns linked to the high-standing volcanoes exhibit high intensity (up to 1500 nT) and elongated, better-organized configuration (quasi-linear spreading associated to moderate extension and high-angle faults; i.e., ODP drill-sites 655 and 651).

Oceans are thought to originate following continental rupture. Oceanic crust with MORB affinity forms in oceans and marginal seas. Basaltic crust accretion from inception to ending may be investigated straightforwardly in small basins rather than in ocean. Two episodes of hyperextension and E-MORB volcanism characterized the Tyrrhenian intra-orogenic basin opening^1,2^. The small oceanic basins of Vavilov and Marsili are located in the central part of the sea (Fig. 1) overlying west-directed subduction of lithosphere belonging to Adria/Ionian microplate. In and around the bathyal area are present stretched remnants of orogenic accretion exhibiting Hercynian, Alpine and Apenninic age. Rifting of Hercynian Corsica-Sardinia and inherited orogen of Alpine age was initiated in early Oligocene^3,4^. Burdigalian-age spreading (~20–16 Ma) of Sardinia-Provence oceanic basin accompanied the counter-clockwise rotation of the adjacent continental blocks of hercynian and alpine age. The Tyrrhenian orogen was produced by SE-directed, *flat-slab* subduction of lithosphere located to the east of Corsica-Sardinia foreland^5,6^. Orogenic accretion was followed by rifting which was in turn supplanted by convergent tectonics reflecting inversion of subduction polarity^3^. The W-directed, *steep-slab* type of subduction was linked to E-verging Apenninic thrusting, back-arc opening and slab migration. The *flat-slab* subduction produced alpine double vergence^3,5,6^ (Fig. 2). Eastdipping front- and westdipping back-thrusts crop out on the western and eastern margins (NE Corsica and Calabrian arc), respectively. Geophysical investigations, deep drilling results of the Deep Sea Drilling Project (DSDP) Leg 42 and of the Ocean Drilling Program (ODP) Leg 107, and seafloor sampling indicate that Vavilov and Marsili bathyal plains are floored by basalts and exposed ultramafic rocks, and were affected by rapid subsidence^4,6–12^. Since the basins’ magnetic anomaly field (Fig. 3) shows significant intensity but does not have the linear symmetric patterns typical of the ocean floor, the intra-orogenic Tyrrhenian opening can not be recognized in a straightforward way on the basis of magnetic signature^13,14^. Thus, in spite of young age (< 8 Ma) the Tyrrhenian spreading process remains poorly documented. In order to contribute to the comprehension of such process we have examined the relationships between (i) extensional tectonics and volcanism in bathyal area, and between (ii) this volcanism and volcanism developed on the conjugated continental margins.

## Episodes of magma-poor hyperextension of initial oceanization

### Late-Tortonian/Early-Messinian (~7.5 – ~6.3 Ma)

Eastwards from the Sardinia island, ~180 km of stretched Hercynian crust form the Tyrrhenian passive margin. Strong asymmetric deformation was produced by east-dipping, low-angle detachment faults which were associated to late Miocene sediments^6^. Inspection of seismo-stratigraphic data (documented in Fig. 4 of ref.^6^) shows that N-S trending listric normal faults reduced from ~30 to 10 km the thickness of continental crust of submerged Sardinian margin, converging basinward with Vavilov plain serpentinized upper mantle (see Supplementary Information). At the plain’s eastern edge, DSDP Site 373 (Fig. 1) penetrated 190 m of basaltic crust; flows and breccias show MORB nature and K/Ar whole-rock age between ~7.5–6.3 Ma^3,8,15^. This dating indicates temporal overlap between Vavilov basin magmatism and hyperextension on the conjugated Sardinian margin without accompanying volcanic activity.

**Figure 4:**
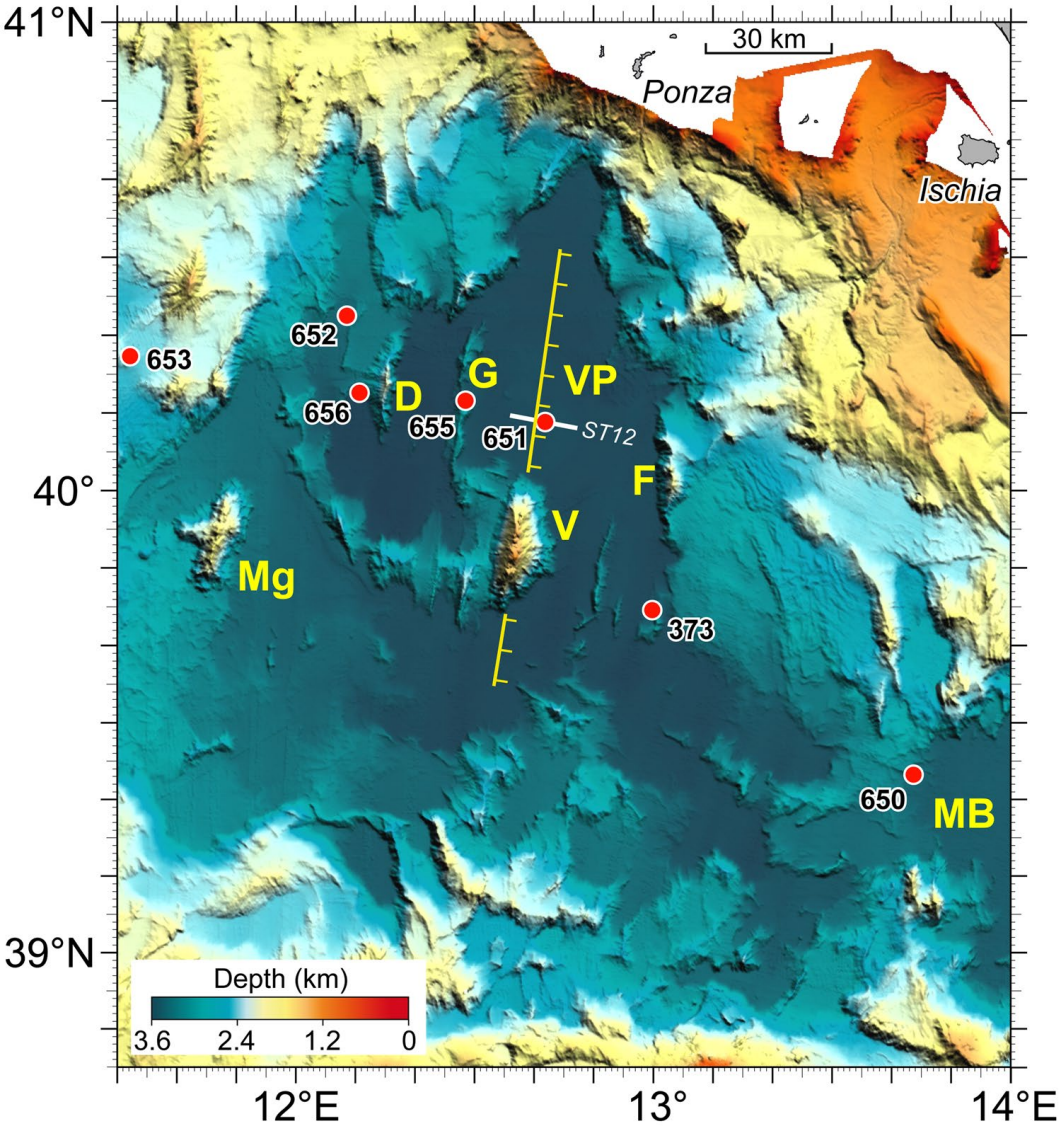
Morphology of the Vavilov Basin. Shaded relief image of Vavilov plain. Data^48–50^ and methods^51–54^ as in Fig. 1. It shows the N-S tectonic line linking Vavilov volcano with the peridotite swell drilled at site ODP 651 (see Fig. 6). G = Gortani ridge, 655 = site of ODP 655 drilling; D = De Marchi seamount; F = Flavio Gioia seamount; M = Magnaghi volcano; MB = Marsili Basin; Pa = Palmarola island; V = Vavilov volcano; VB = Vavilov basin. Location of seismic profile ST12 shown in Fig. 6 is indicated by a white solid line. Location of ODP 650 and DSDP 373 drillings are at the western and eastern edges of Marsili and Vavilov plains, respectively.

Such tectono-magmatic evidence indicates that late Miocene basalt crust formation of Vavilov basin preceded the Mediterranean salinity crisis (late Messinian; 5.96–5.33 Ma). The absence of evaporitic deposits in bathyal area is probably due to existing shallow water conditions^3,8^. Emplacement of MORB melts and exposure of serpentinized peridotite (ODP site 651) in the Vavilov basin, and hyperextension at the submerged Sardinian margin appear to recall the tectono-magmatic architecture linked to the Mesozoic continent–ocean transition at the Iberian margin^6,16^.

### Latemost Pliocene (~1.9 – ~1.7 Ma; Olduvai subchron)

**Figure 5:**
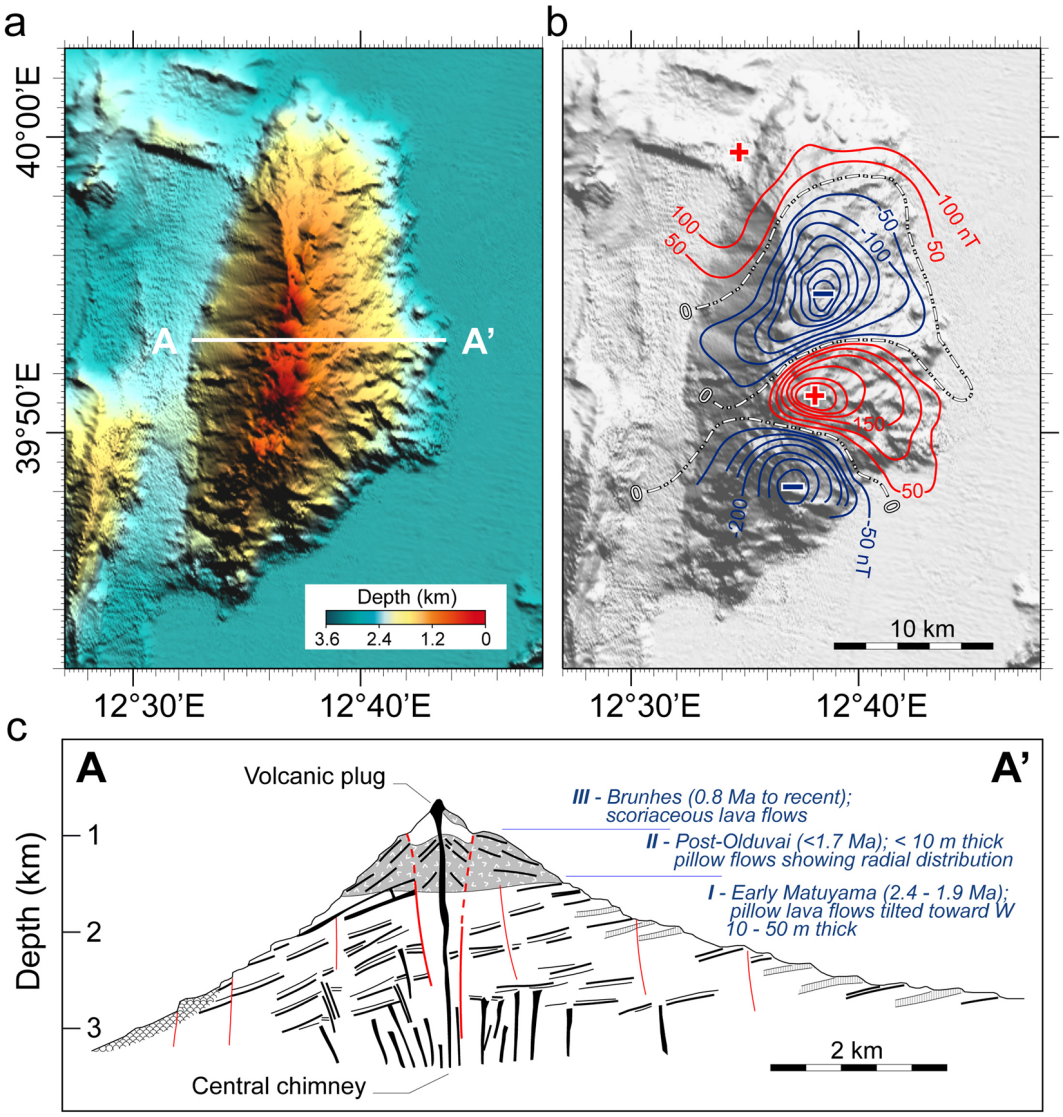
The Vavilov volcanic complex. (**a**) Colour-shaded relief image based on multibeam data from ref.^48^. The multibeam data were processed by the Kongsberg Neptune/Poseidon packages; spatial analysis and mapping were performed using the GMT^51^ and PLOTMAP^52^ packages. Thick white line indicates the location of the cross-section shown in (**c**). (**b**) Gray-shaded relief with over-imposed contour lines (contour interval of 50 nT) of the shipborne magnetic anomaly field. Magnetic anomaly contours were digitized in geographical coordinates from ref.^11^ by using the software DIGMAP^53^ and were converted from the ED50 to the WGS84 geodetic datum by the program DATUM^54^. Red and blue contour lines indicate positive and negative magnetic anomalies, respectively, while zero level contour is marked by dashed-dotted line. (**c**) W-E cross-section of Vavilov volcano based on submersible observations from 3000 m depth to the top 684 m below sea level (see text). The summit ridge is formed by two principal eruptive centres. Vavilov volcanic complex rises from the bathyal plain at 3600 m b.s.l., and is 35 km in length and 15 km in width. (**c**, inspired by ref.^18^).

**Figure 6:**
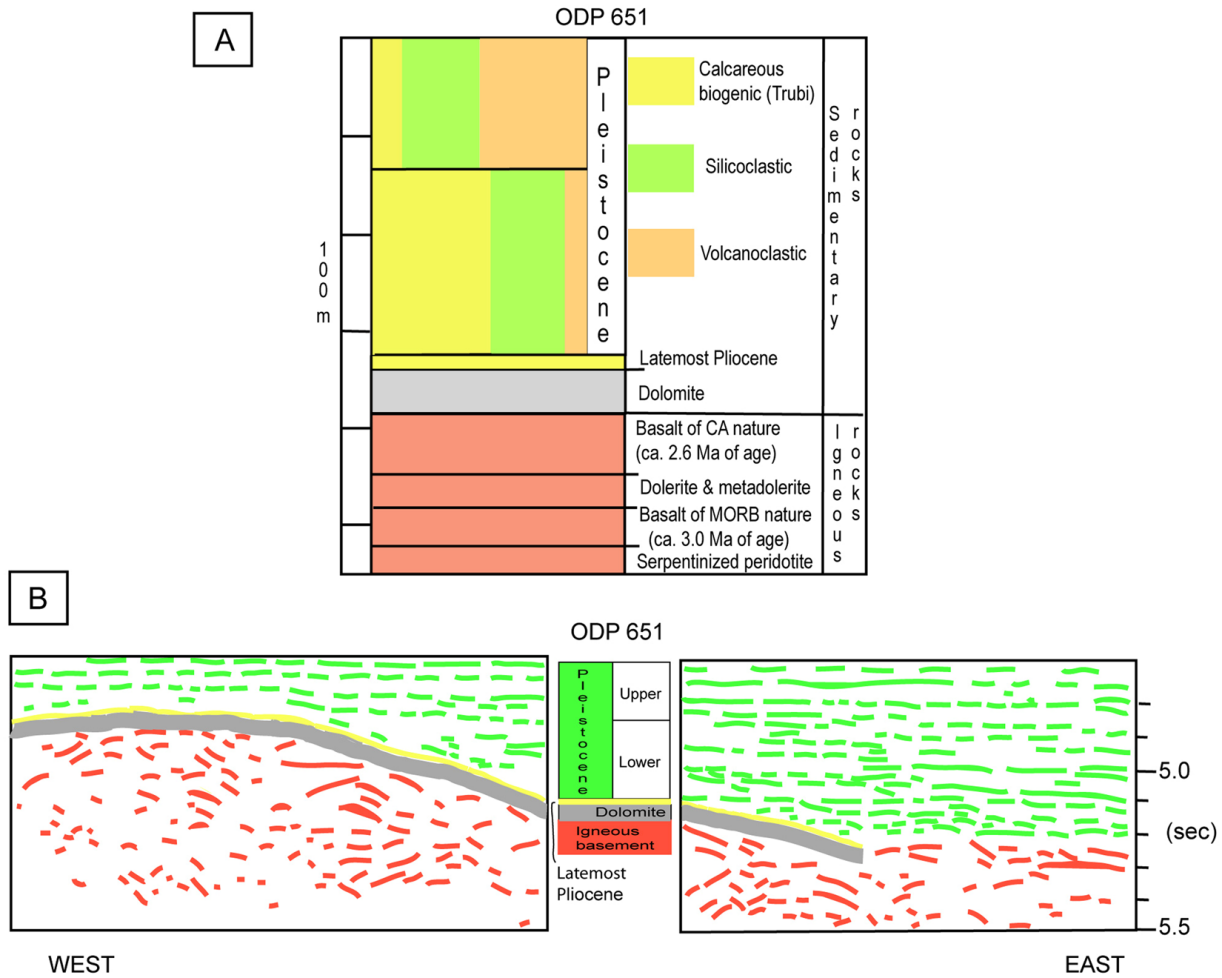
Stratigraphy of ODP Site 651 (Vavilov plain). (**A**) Main litho-stratigraphic units; the lower unit consists of complex igneous basement which has serpentinized peridotite at the bottom, whereas sediments of LmP and Pleistocene age form the upper unit. (**B**) Line drawing of the seismic reflection profile ST12 crossing drill-site on east flank of basement swell (see text). Location of seismic reflection profile is indicated in Fig. 4.

In the axial zone of Vavilov basin, inherited alpine orogen, the N-S elongated tectono-magmatic lineament linking Vavilov-volcano to exposed-peridotite^8^ is associated with lineated magnetic anomalies (Figs 3–5). The northern portion shows positive anomaly with intensity up to 70 nT (airborne survey)^13^. Intensity of 200 nT was obtained from shipborne data from the same area^11^. The southern portion of the lineament is characterized by alternating negative and positive values of magnetic field (Fig. 5b). Seismostratigraphic evidence suggests that this tectonic lineament is due to detachment faulting^8^. ODP Site 651 drilled through the faulted basement swell which consists of peridotites, dolerites and basalts. Such basement complexity is overlain by thin late Pliocene ooze (*Globorotalia inflata* zone; MPl-6; 2.6/2.4 – 1.9 Ma) which is below 340-m-thick Pleistocene sedimentary sequence (Fig. 6). Positive magnetization and foram assemblage of ooze directly overlying basement indicate that east-dipping detachment linked to mantle exposure occurred in the latemost Pliocene (LmP) - Olduvai subchron, ~1.9–~1.7 Ma. The classical scales of geological time contemplate that the Olduvai corresponds to the Tertiary/Quaternary boundary (~1.8 Ma) which separates the Pliocene from the Pleistocene portion of Matuyama chron. Based on seismostratigraphy evidence (Fig. 6), the peridotitic body was exposed through slip on east-dipping detachment fault acting in LmP time. Probably coinciding low-angle extensional faulting acted at Vavilov seamount too (Fig. 5c), without accompanying volcanic activity. Magmatism linked to the volcano-peridotite lineament is an issue of the next chapters.

At the same time of Vavilov plain hyperextension, oceanic opening was initiated in Marsili basin (Fig. 4) coinciding with volcanism absence on the emerged conjugate margins. At western edge of Marsili igneous basement (Fig. 4) ODP Site 650 drilled basaltic crust with MORB transitional to calcalkaline affinity. The basalt lava is linked to round shape, positive magnetic anomaly with intensity up to 200 nT^11^. The forams assemblage and the positive magnetization of ooze (Trubi) directly overlying the igneous basement indicate LmP age, as at ODP Site 651. The overlying Pleistocene sequence consists of 600-m thick, sediments of terrigenous and volcanoclastic nature^8,9^. Being strongly altered and vesicular, the early basalt flows of Marsili plain probably erupted in shallow marine environment.

## Episodes of ocean-floor moderate tensional tectonics and seamount volcanism

### Pliocene (~5 – 1.9 Ma)

The N-S trending Gortani ridge was drilled at ODP Site 655 (Fig. 4). 116 m-thick basalt flows beneath only 80 m of Pliocene-Quaternary sediments show MORB affinity and ^40^Ar/^39^Ar age of 4.3 Ma^10^. To the west the Gortani ridge, basaltic crust is flanked by N-S elongated sedimentary basin showing > 1 sec sediment thickness^6^. With respect to MORB volcanism from DSDP Site 373, Gortani’s location is more distant from the W-dipping front of Adria-Ionian subduction. Such remoteness is probably a consequence of (i) past shallow depth of subducted lithosphere with little slab pull towards the active margin and/or (ii) the hyperextension that affected bathyal area and Sardinia submerged margin in late Miocene allowed Pliocene MORB volcanism migration towards the passive margin. After the Gortani, such volcanism migrated only towards the hinge zone.

ODP-651 drilling is located ~20 km to the east of Gortani ridge, and 15 km to the north of Vavilov volcano along the volcano-peridotite tectonic line, at the axis of Vavilov basin. In the northern side of lineament, serpentinized harzburgites were drilled for 30 m at the base of the well. They rest below a 135-m-thick sequence of basalt flows separated by an intrusive dolerite body^8^ (Fig. 6). The Site’s lower basalts of MORB composition yielded ^40^Ar/^39^Ar dating of 3.0 Ma^10^. The upper basalts have calc-alkaline affinity and about 2.6 Ma of age. Such datings indicate the Gauss chron for the N-S elongated positive anomaly with maximum intensity of 200 nT at ODP Site 651^11^. Figure 6 shows that igneous basement is overlain by 40-m-thick sterile dolostone^17^ which rests directly beneath LmP ooze (see previous chapter). On the other side of lineament, the magnetic anomaly field associated with Vavilov volcano shows negative and positive values^11^ (Fig. 6). On the basis of shipborne measurement, the northern and southern negative magnetic anomalies exhibit values up to −430 and −360 nT, respectively. The intervening positive zone with maximum intensity of + 370 nT is close to the seamount’s top. It is W-E elongated whereas the “peak-values” of normally and inversely magnetized bodies are NNE-SSW aligned with the relief axis. Seabeam bathymetry and submersible dives (Fig. 5) reveal that the west flank of Vavilov is significantly steeper than that to the east^18,19^. Figure 5 shows that Vavilov volcanism is divided in lower, intermediate and higher units. The lower unit (I) at waterdepth between 3.600 and 1.500 m is linked to negative anomaly. It erupted in the Pliocene portion of Matuyama chron – between 2.6/2.4 and 1.9 Ma. In fact, “Trubi-type” ooze from the western flank at 2880 m depth contains foram assemblage showing late Pliocene age. The rock was thermally indurated on contact with hot lava^18^. Massive pillow and bolster flows form flow-fronts that are up to 50-m-high. On the eastern flank, the basalt scarps are next to sediment-covered shelves up to 250-m wide. Flows and shelves of this flank were manifestly tilted towards the west and stretched towards the east. Their inclination towards the volcano’s axial zone was likely produced by the same east-dipping detachment faulting that exposed the peridotite swell at ODP Site 651 in LmP time (~1.8 Ma). So, the intermediate unit (II) at waterdepth of 1500–1000 m probably erupted in post-Olduvai time (<1.7 Ma). It consists of pillow flows that have <10-m-thickness and equal dip at either flank of seamount. Scoriaceous lavas of alkaline (intra-plate) nature form the upper unit - discussed below.

### Quaternary (~1/0.8 Ma to Recent)

In the volcano-peridotite lineament of Vavilov basin, volcanic activity developed also in the late Pleistocene after the Matuyama/Brunhes geomagnetic reversal (<0.8 Ma)^20^. Figures 3 and 5 show the N-S elongated Matuyama’s negative magnetic anomaly of Vavilov volcano which is interrupted by circa E-W elongated positive anomaly^11^. It is located near to the top of seamount and it widens eastwards. The NNE-SSW aligned “peak-values” of normally and inversely magnetized bodies parallel the relief axis. Basalts from the upper unit are most likely linked to the Brunhes chron^15^. They exhibit alkaline composition and K/Ar age of ~0.4 and <0.1 Ma^18,21^. Alkaline basalts form by partial melting of mantle sources which are compositionally distinct from sources which experienced metasomatic modifications from subducted lithosphere at the origin of calcalkaline melts. Despite the scrupulous dives of Cyana and Mir submersibles and numerous dredge hauls, the fresh pillow flows forming the bulk of Vavilov seamount did not yield any specimen. A reason for this may be that lava detritus is overlain by sediment. Given the diversity of morphology, age and magnetic field, the chemistry of lower unit and summit lavas is probably different.

Volcanism from large Marsili seamount appears to be not older than 0.8 Ma based on the Brunhes chron and the < 0.2 Ma age of rocks dredged from the summit zone^11,15,22^. The seamount magnetic anomaly field, on a small scale shows transition from a lateral spreading to a pure vertical growth which probably initiated at the Jaramillo subchron^23^. The recovered samples constantly show calc-alkaline affinity; high-potassium andesites are present at the summit, whereas basalts from the intermediate-deep zone show medium- to low-K composition^24^. Voluminous volcanism of calc-alkaline affinity of the Aeolian arc developed since ~1 Ma around Marsili plain^1,3^ from magma sources metasomatized from subducting lithosphere.

## Volcanism on the conjugated continental margins of Sardinia and Campania; their relationships with bathyal volcanism

### The Rifted Continental Margin from Sardinia

#### The orogenic (calcalkaline) cycle

**Figure 7:**
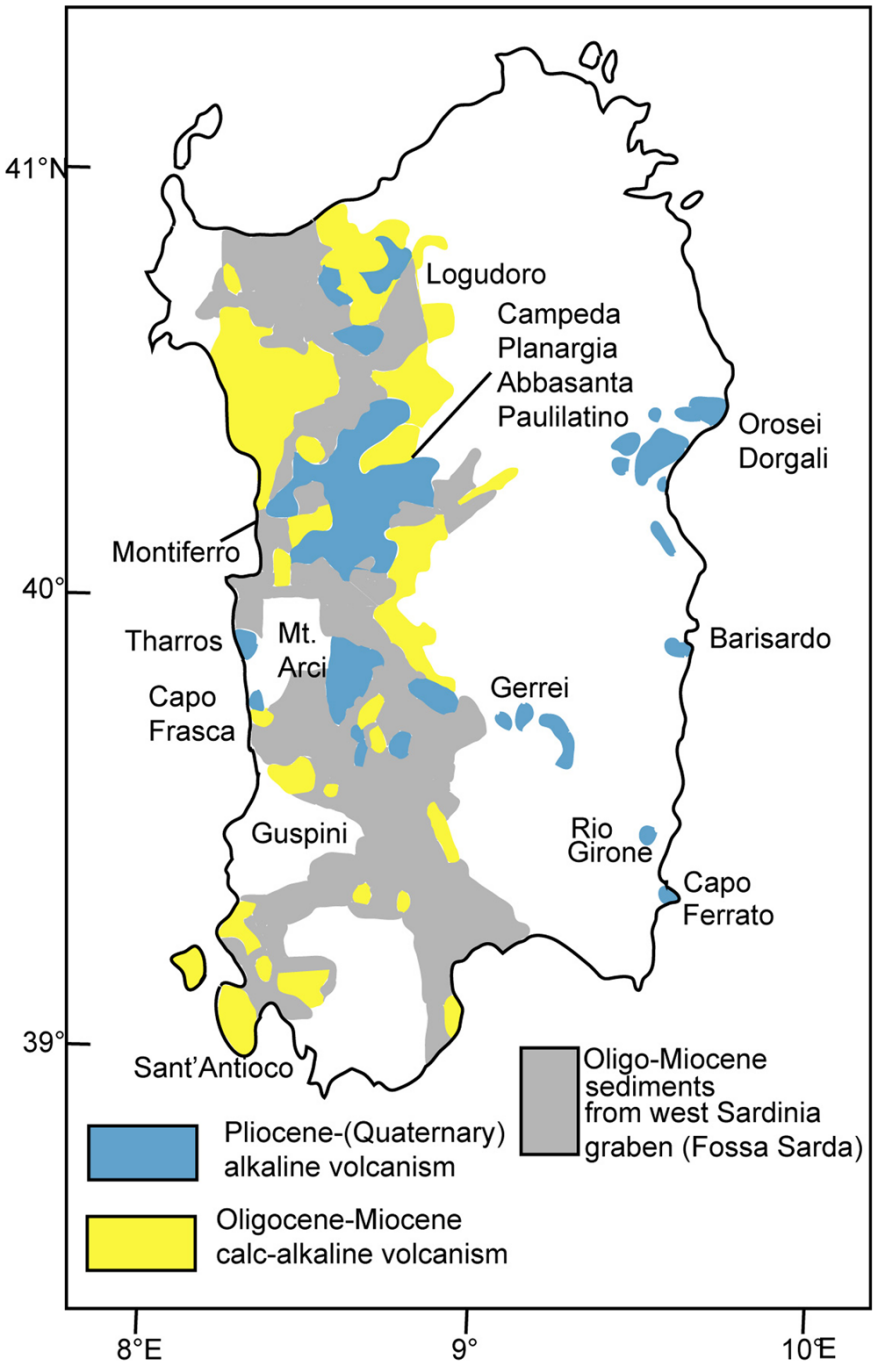
The volcanic cycles from Sardinia. Simplified map showing that Sardinian volcanism developed in Oligocene-Miocene and Pliocene-(late Quaternary) time, and that pre-Pliocene sediments are found only in the “Fossa Sarda” of western Sardinia (see text). Mapping was carried out using PLOTMAP^52^ software with boundaries of volcanic provinces from refs^25,29^ digitized in geographical coordinates by using DIGMAP^53^ software and converted to the WGS84 geodetic datum by DATUM^54^ program.

Since early Oligocene the Hercynian island of Sardinia was affected by two distinct cycles of volcanic activity with an interruption lasting ~7 Ma (Fig. 7). The Oligo-Miocene cycle of calcalkaline affinity (~30–12 Ma) developed in the N-S trending trough of western Sardinia^25,26^. The “Fossa Sarda” was affected by rifting concomitantly with rifting of Provence conjugated margin until ~20 Ma. At this time, continental rift reached the stage of oceanic opening of Sardinia-Provence basin and rotation of the Corsica-Sardinia block (~20–16 Ma)^3,27^. Andesites and ignimbrites are the main manifestations of the Oligo-Miocene volcanism. Pillow-lavas with tholeiitic, high-Mg basalt composition are associated with early Burdigalian (~20–18 Ma) shallow marine sediments from central western Sardinia^28^. It appears that effusion of mafic volcanics in Sardinia (~20–18 Ma) was coincident with oceanic opening of Sardinia-Provence basin.

#### The anorogenic (alkaline) cycle of Pliocene-(late Quaternary) age

The subaerial anorogenic (within-plate) cycle initiated to develop only after opening of Vavilov plain. The Pliocene basalt volcanism with varying alkaline to subalkaline composition is found in both western and eastern Sardinia^29^ (~5/4–2 Ma; Fig. 7). It was related to tensional tectonics which involved also Tyrrhenian seafloor. Subaerial basalt flows bordering the Orosei Gulf have been argon dated between 3.6 and 2.0 Ma^30^. In the distant Gulf offshore (Fig. 1), by the edge of bathyal plain ~75 km west of Vavilov seamount is the location of Magnaghi seamount. Magnaghi’s alkaline basalt lava shows ~3.0–2.7 Ma^31^ which recalls the age of peak alkaline basalt volcanism from Sardinia (~2.8 Ma^21,29^). The Magnaghi rocks represent the only known manifestation of Pliocene volcanism from the Sardinia continental slope. The within-plate cycle from Sardinia ceased to develop between ~2–0.9/0.8 Ma. The early part of this period (~1.9–1,7 Ma) saw strong extensional tectonics and initial oceanic opening of Vavilov plain and Marsili plain, respectively. The anorogenic volcanism continued to develop in late Quaternary (~0.9/0.8–0.1 Ma). Yet, it is found only in the Logudoro region (i.e., NW Sardinia), and without accompanying manifestations with subalkaline characteristics^21,32^. The Logudoro “basalti delle valli” and the rocks from the summit area of Vavilov volcano were related to late Quaternary tensional tectonics.

### The offshore Campania margin

**Figure 8:**
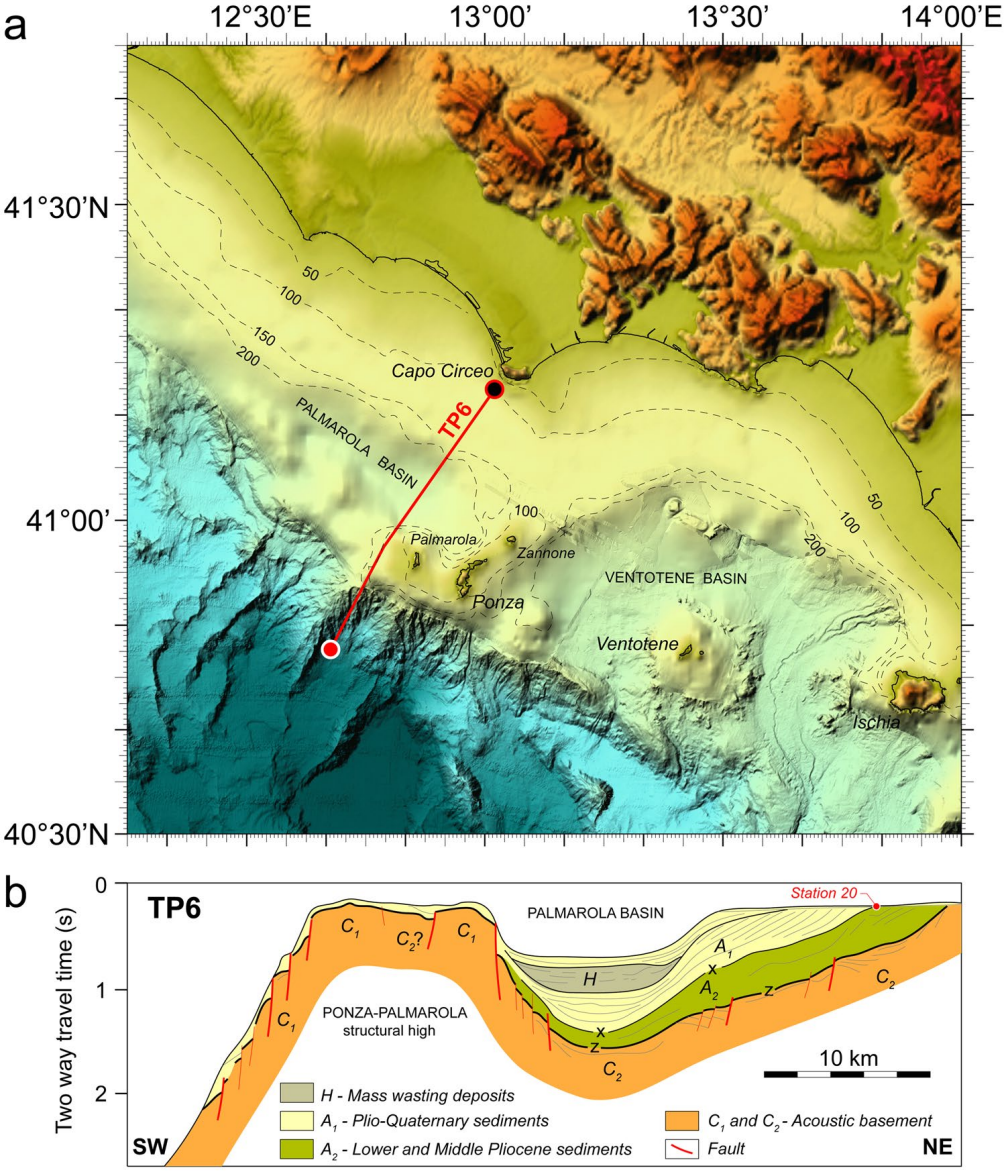
The Ponza structural high. (**a**) Bathymetric map showing Latium-Campania offhore, the volcanic structural high of Palmarola Ponza and Zannone islands, and the adjacent sedimentary basins of Palmarola, Ventotene and Vavilov. Data^48–50^ and methods^51–54^ as in Fig. 1. (**b**) Line drawing and interpretation of seismic reflection profile TP6 across Palmarola basin; early Pliocene sediment is found at coring station n. 20. TP6 profile shows that Capo Circeo offshore and Palmarola basin have been submerged only shallowly until early Pliocene. Palmarola basin formation and calcalkaline rhyolitic volcanism from Ponza island started in early Pliocene (4.4 Ma). Legend. *Seismic unit A* – Plio-Quaternary sedimentary sequence divisible in: unit A1, sedimentary sequence from the mid-Pliocene to the present including seismic unit H; unit A2, sedimentary sequence including lower and middle Pliocene. *Seismic unit C* – acoustic substratum divisible in: unit C1, non reflective; unit C2, reflective. X: middle Pliocene unconformity; Z: top of the acoustic substratum (see text). (**b**, inspired by ref.^34^).

Seaward from Campania three sedimentary basins border on the structural high of Ponza Palmarola and Zannone islands which form the western portion of Pontine Archipelago (Fig. 8)^4,33,34^. To the south, very steep slope directly connects Palmarola with the bathyal domain (Fig. 4). Conversely, the basins from Palmarola and Ventotene, positioned respectively NW and SE of the structural high, are parallel to Campania coast. Ponza Ventotene and Palmarola to the west, and Ventotene and S. Stefano to the east are entirely volcanic islands, whereas at Zannone crop out also metamorphic rocks (phyllites) and sediments showing Mesozoic - Tertiary age^4,35^. Pontine volcanism has episodic nature^33,36,37^. ~4.4 Ma old rhyolites of calcalkaline affinity and ~1.1 Ma K-rich trachytes extruded at Ponza. Ponza trachytes, and 0.8-Ma-old basalts from Ventotene and S. Stefano islands are early products of the late Quaternary Roman Magmatic Region^38,39^. More to the east, significant K-rich volcanism of the last 0.2 Ma developed in connection with tensional and transtensional faulting in and around Ischia and Procida islands (Campanian shelf), and Phlegrean Fields and Vesuvius (Naples bay)^40^. The extrusion dome of Palmarola ~1.75 Ma of age (LmP) is made of rhyolites of transitional composition between calcalkaline and alkaline^33,41^. Palmarola rocks appear to overlap temporally the hyperextension along the lineament linking Vavilov-volcano to exposed-peridotite. Early Pliocene calcalkaline rhyolitic volcanism, and normal and wrench extension faulting initiated to form the Palmarola and Ventotene basins^34^. Based on seismic reflection profiling and geological data, a widespread middle-Pliocene sedimentary unconformity was recognized on the Tyrrhenian margins, and it was considered to reflect overall basin-wide subsidence^42^. In Vavilov plain, Gortani ridge volcanism and adjacent basin formation developed in the early Pliocene **(**~4.3 Ma) shortly before large-scale unconformity occurrence^6,10^ (Supplementary Information). Gortani’s localized tectono-magmatic manifestation has probably put an end to late-Miocene seafloor shallowness of Vavilov basaltic crust. In the Campania shelf, landward from Ventotene basin a late-Miocene to Quaternary W-E trending submerged sedimentary sequence has been recognized^34^. The paleo-basin of Terracina, “presently masked by the continental shelf shows a marginal, terrigenous facies type surely lacking the evaporitic rocks so characteristic of some Tyrrhenian margins”^34^. With time, the late Miocene W-E distensive deformation from Gaeta gulf was supplanted by NW-SE extensional tectonics and volcanic activity compatible with the youngest extension direction of Tyrrhenian basin^4,40^.

## Discussion

### The basalt - peridotite lineament of Vavilov plain

Regarding the nature of Tyrrhenian igneous basement, some authors^12^ suggest that the area floored by peridotite is larger than that showing basalt compostion^6^. From a geodynamic perspective, the former view may imply that Tyrrhenian opening was mainly forced by extensional tectonics and the latter by magmatic activity. The magmato-tectonic lineament linking Vavilov-volcano to peridotite might abut the volcanic island of Palmarola (Figs 4 and 8). LmP hyperextension along the Vavilov plain tectonic line accompanied by rhyolitic volcanism from Palmarola might announce inception of Vavilov-Marsili basaltic crust rapid subsidence. Such event was preceded by basin formation linked to high-angle faulting affecting basaltic plain and margins’ continental crust. Pliocene to Quaternary sedimentary basins are found only in submerged Sardinia margin, and in subaerial and submerged portions of Campanian margin, and may be also elsewhere^6,34,43^. On the Sardinia margin, ODP Site 654 drilled lava flow interbedded in Pliocene-Quaternary sedimentary sequence^10^ (Fig. 1). It is a basaltic andesite of LmP age, and is analogous to some subaerial Pliocene manifestations of intraplate affinity from Sardinia^29^. Figure 1 suggests that NW-SE trend of LmP oceanic opening supplanted the late Miocene E-W extension linked to basaltic crust formation of Vavilov plain. In the LmP episode, volcanism was localized only in marine areas. On the emersed margins of Sardinia and Campania the volcanic activity resumed to develop only about 1/0.9 Ma ago.

N-S elongated sedimentary basin showing > 1 s sediment thickness formed to the east of the LmP lineament^6^ (Supplementary info). Location of lineament is in the central basalt plain of Vavilov amid De Marchi and Flavio Gioia seamounts (Fig. 4). Crystalline-metamorphic basement is found at either relief. The Flavio Gioia is composed by late Hercynian granitoids and metamorphic rocks of various grade involved as tectonic units of the Alpine orogen; this crystalline-metamorphic association recalls the back-thrusts from Calabrian arc^44^. Conversely, the crystalline-metamorphic De Marchi relief includes tethyan ophiolites. They have been sampled in several other Tyrrhenian sites and appear to be similar to the front-thrust ophiolites from Alpine Corsica^6,45^. From a structural point of view, the tectonic line probably represents the collapsed geosuture linked to past double verging alpine-age crustal accretion (Fig. 2). Back-thrusted allochthons of metamorphic basement are present also in the internal side of Apennines^46^. The Apennines are characterized by clearly distinct modes of tectonic activity. Extensional and compressional deformation affect the thrust belt internal (Tyrrhenian) and external (Adriatic) side, respectively. Also the seismicity focal aspects of compressional and extensional type mirror the tectonic mode diversity at either Apennines side^47^. Extensional tectonics of the calcareous reliefs from Monti Sibillini and surroundings was at the origin of strong earthquakes and prolonged aftershock sequences from August 2016 to January 2017.

### Evolution of volcanism and magnetic anomaly field

The anomalies associated with deep-seated basaltic crust have low intensity (up to 200 nT^11^) and shapes which are almost rounded or poorly elongated (diffusional spreading linked to strong extension and low-angle faults (the drillsites DSDP 373 and ODP 650; see inset -a- of Fig. 3). By contrast, the magnetic patterns linked to the high-standing basaltic crust (volcanoes) exhibit high intensity (up to 1500 nT) and elongated, better-organized configuration (quasi-linear spreading associated to moderate extension and high-angle faults). The elevation of elongated edifices strongly increased moving eastwards. Gortani ridge, Vavilov seamount and the over-fed Marsili seamount may be considered to be a “sui-generis” southeastward migrated sequence of N-S oriented axes of spreading.

## Conclusions

–The Tyrrhenian opening was initiated in the late Miocene before Messinian salinity crisis and margins’ volcanism. Strong extensional deformation (drifting) was a consequence of E-directed low-angle faulting of the Sardinia offshore margin accompanied by ascent of MORB melts at the shallow eastern edge of Vavilov plain (DSDP-Site-373). Subsequent, E-directed low-angle faulting along the magmato-tectonic lineament linking Vavilov volcano to exposed peridotite acted in LmP time (~1.9–1.7 Ma). The short-lived hyperextension along Vavilov axial zone experienced concomitant opening of Marsili plain linked to emplacement of basalt melts (ODP-Site-650 at the plain western edge) without accompanying volcanism on the emerged margins.

–The post drift, Pliocene (~5/4 – 1.9 Ma) and late Quaternary (<1 Ma) basalt crust formation was characterized by moderate extension, high-angle faulting (rifting) and voluminous volcanism. Concomitantly, Pliocene volcanism developed in bathyal area and on conjugated continental margins. Early Pliocene (~4.4 Ma) MORB lava at ODP site 655 and basin formation from Vavilov plain^6,10^, and rhyolites from Ponza island and basin formation at Palmarola (submerged Campanian margin)^33,34^ probably indicate starting evolution from shallow to deep water conditions of the mafic crust.

–From a structural point of view, the volcano-peridotite lineament might represent a geosuture produced by alpine-age crustal accretion that probably implied obduction of large ultramafic bodies^12^. In this view of peridotitic lineament, the terms exposure/exposed can be be more appropriate than exhumation/exhumed. The lineament was affected by episodic volcanic activity from early Pliocene (ODP-Site-651; ~3.0 Ma) to late Quaternary. It likely abuts the structural high of Ponza archipelago. The extrusion of Palmarola rhyolites and hyperextension of Vavilov plain (~1.75 Ma) possibly triggered basin-scale extension change from W-E to NW-SE direction and inception of basaltic crust rapid subsidence^4,33,40^. Extensional tectonics and basalt volcanism of Tyrrhenian opening reflect crustal stretching of inherited alpine-age orogen, being itself related to rifting of the Apennines internal side.

Nevio Zitellini and CS produced the PDF files of article^34^ and articles^21,24,25,29,30,33,37,38,44^, respecticely. Such PDF files are found in the authors’accounts at www.researchgate.net - ResearchGate is probably the only way to obtain all this info.

## Electronic supplementary material

Supplementary information
