# Supplementary material for: An updated reconstruction of basaltic crust emplacement in Tyrrhenian sea, Italy
Source: Sci Rep. 2017 Dec 21;7:18024. doi: 10.1038/s41598-017-17625-2 (PMC5740153; doi:10.1038/s41598-017-17625-2)
Supplement: Supplementary file 1 — Supplementary information [file 41598_2017_17625_MOESM1_ESM.pdf]

SUPPLEMENTARY INFORMATION

AN UPDATED RECONSTRUCTION OF BASALTIC CRUST EMPLACEMENT IN TYRRHENIAN SEA, ITALY

Carlo Savelli[(1)] and Marco Ligi[(2)]
[(1)]Marine Geologist (retired from Italian CNR)
[(2)]Institute of Marine Sciences (Geology) - CNR

Hyperextension of the continental margin offshore Sardinia and Vavilov plain volcanism

The seismic line drawings presented in the figure 4 created by *Sartori et al*. 2004 (main article reference[6]) show that low-angle, east-dipping faults produced strong asymmetric extension of Sardinia passive margin. According to the seismo-stratigraphic reconstruction strong extension tectonics acted in the late Tortonian/early Messinian. Concerning the age of volcanism linked to early formation of Tyrrhenian basaltic crust and the extrusion of MORB basalts at DSDP Site 373, the authors[6] proposed the Pliocene (< ~5 Ma), and this article the late Tortonian/early Miocene (~7 Ma) which coincides with the hyperextension pulsation. So, in the former view basaltic crust was emplaced after and in the latter before the late Messinian salinity crisis. Both views acknowledge the absence of evaporitic rocks in the Tyrrhenian central area of basaltic nature. Though, one view considers that water conditions of the oceanic crust by the end of Messinian were not yet deep (see main article text) and the other one that they were already deep (*Sartori et al*. 2004).

Re-observing figure 8 by *Sartori et alii* - 2004

The comprehensive figure 8 was created by Authors[6]. It show the distribution of Pliocene-Quaternary sediment thickness. In the basaltic crust of Vavilov plain, NS trending and >1 sec thick sedimentary basins were formed at the western flank of Gortani ridge as well as to the east of the magmatic lineament linking Vavilov basalt seamount to peridotite drilled at ODP site 651 and to rhyolites from Palmarola island (see also figure 4 from main article). Local basin formation and its relationships with Pliocene-Quaternary volcanism are discussed in article text.
In their figure 8 Authors[6] recognize the presence of volcanic bedrock located between De Marchi seamount and the NE-SW oriented physiographic lineament at western edge of Vavilov plain (to the south of ODP Site 652; see also figure 4 from main article). Such bedrock (unit 5b from figure 8 legend) has been interpreted to be a subvolcanic body[6] emplaced between 5 and 4 Ma (Pliocene). Alternatively, the bedrock may be hypothesized to represent a foundered volcano of Oligocene-Miocene age. In fact, in the inherited Tyrrhenian orogen of Alpine age calcalkaline volcanism probably developed from early Oligocene to mid Miocene[3]. In this view, *lost volcanoes* were at the origin of the eruptions that produced the allochthonous volcanoclastic rocks of the Apennines. Oligocene to mid Miocene volcanism was linked to rifting of continental crust thickened by Alpine-age orogenic accretion. Pre-oceanic rifting of continental crust made possible the post-late-Miocene quick opening and subsidence of Tyrrhenian bathyal area.
